# Supplementary material for: Trajectories of recall memory as predictive of hearing impairment: A longitudinal cohort study
Source: PLoS One. 2020 Jun 18;15(6):e0234623. doi: 10.1371/journal.pone.0234623 (PMC7302912; doi:10.1371/journal.pone.0234623)
Supplement: S1 Table — (DOCX) [file pone.0234623.s001.docx]

**Supplementary Table 1:** Comparisons of the excluded and analytic sample in Wave 7

| **Variable** | **Excluded sample**  **N=726** | **Analytic sample**  **N=3,615** | **p-value** |
| --- | --- | --- | --- |
| Objective hearing impairment | 195 (26.8) | 257 (6.9) | <0.001 |
| Episodic memory score | 8.99 (3.8) | 10.2 (3.6) | <0.001 |
| Age | 74 (7.2) | 72.2 (6.9) | <0.001 |
| Female | 308 (42.4) | 2,158 (59.7) | <0.001 |
| *Marital status* |  |  | 0.658 |
| Single | 32 (4.4) | 165 (4.5) |  |
| Married | 450 (61.9) | 2,241 (61.9) |  |
| Divorced | 76 (10.4) | 425 (11.7) |  |
| Widowed | 168 (23.1) | 784 (21.6) |  |
| *Education* |  |  | <0.001 |
| Primary | 276 (38) | 963 (26.6) |  |
| High school | 117 (16.1) | 722 (19.9) |  |
| College or higher | 333 (45.8) | 1,930 (53.3) |  |
| *Wealth* |  |  | <0.001 |
| 1^st^ tertile (poorest) | 275 (37.8) | 1,096 (30.3) |  |
| 2^nd^ tertile | 249 (34.3) | 1,260 (34.8) |  |
| 3^rd^ tertile (richest) | 202 (27.8) | 1,259 (34.8) |  |
| *Smoking Behaviour* |  |  | 0.001 |
| Non-smoker | 275 (37.8) | 1,547 (42.7) |  |
| Past smoker | 367 (50.5) | 1,782 (49.2) |  |
| Current smoker | 84 (11.5) | 286 (7.9) |  |
| Drinking regularly | 120 (18.9) | 681 (21) | 0.203 |
| Moderate physical activity | 357 (49.1) | 2,197 (60.7) | <0.001 |
| Vigorous physical activity | 76 (13.5) | 642 (17.7) | 0.005 |
| *The presence of chronic diseases* |  |  |  |
| Heart diseases | 76 (10.7) | 223 (6.1) | <0.001 |
| Diabetes mellitus | 116 (15.9) | 475 (13.1) | 0.041 |
| Stroke | 56 (7.7) | 207 (5.7) | 0.041 |
| Cancer | 62 (8.5) | 247 (6.8) | 0.083 |
| Lung diseases | 72 (9.9) | 202 (5.5) | <0.001 |

Note: * Presented are mean (SD) or number (%).
