# Supplementary material for: Trajectories of recall memory as predictive of hearing impairment: A longitudinal cohort study
Source: PLoS One. 2020 Jun 18;15(6):e0234623. doi: 10.1371/journal.pone.0234623 (PMC7302912; doi:10.1371/journal.pone.0234623)
Supplement: S2 Table — (DOCX) [file pone.0234623.s002.docx]

**Supplementary Table 2** Regression parameters of the latent class models

|  | Coefficient | Wald (0) | df | p-value |
| --- | --- | --- | --- | --- |
| Cluster 1 (highest memory) | -0.41 | 542.08 | 3 | <0.001 |
| Cluster 2 (2^nd^) | 0.71 |  |  |  |
| Cluster 3 (3^rd^) | 0.58 |  |  |  |
| Cluster 4 (lowest memory) | -0.89 |  |  |  |
|  |  |  |  |  |
| Recall memory <- 1 | 10.41 | 30615.03 | 1 | <0.001 |
| Recall memory <- Cluster 1 (highest memory) | 4.29 |  | 3 | <0.001 |
| Recall memory <- Cluster 2 (2^nd^) | 1.53 |  |  |  |
| Recall memory <- Cluster 3 (3^rd^) | -1.21 |  |  |  |
| Recall memory <- Cluster 4 (lowest memory) | -4.61 |  |  |  |
|  |  |  |  |  |
| Variances |  |  |  |  |
| Recall memory | 5.60 | 11284.95 | 1 | <0.001 |
