# Supplementary material for: Trajectories of recall memory as predictive of hearing impairment: A longitudinal cohort study
Source: PLoS One. 2020 Jun 18;15(6):e0234623. doi: 10.1371/journal.pone.0234623 (PMC7302912; doi:10.1371/journal.pone.0234623)
Supplement: S3 Table — (DOCX) [file pone.0234623.s003.docx]

**Supplementary Table 3:** Fit criteria for set of latent class models

| Fit criteria | 2 Classes | 3 Classes | 4 Classes | 5 Classes |
| --- | --- | --- | --- | --- |
| Bayesian Information Criteria (BIC) | 118175 | 115725 | 114822 | 114538 |
| Akaike Information Criteria (AIC) | 118107 | 115607 | 114655 | 114321 |
| Class 1* | 1875 (51) | 1700 (47) | 1395 (38) | 1075 (29) |
| Class 2* | 1740 (48) | 1063 (29) | 1388 (38) | 1032 (28) |
| Class 3* |  | 852 (23) | 445 (12) | 1009 (27) |
| Class 4* |  |  | 387 (10) | 270 (7) |
| Class 5* |  |  |  | 229 (6) |

**Note:** * presented as number (%)
