# Supplementary material for: Trajectories of recall memory as predictive of hearing impairment: A longitudinal cohort study
Source: PLoS One. 2020 Jun 18;15(6):e0234623. doi: 10.1371/journal.pone.0234623 (PMC7302912; doi:10.1371/journal.pone.0234623)
Supplement: S4 Table — (DOCX) [file pone.0234623.s004.docx]

**Supplementary Table 4:** Results for hearing function models (with and without recent trajectories of recall memory)

|  | **Model 1** | | **Model 2** | |
| --- | --- | --- | --- | --- |
|  | **Coeff (SE)** | **p-value** | **Coeff (SE)** | **p-value** |
| Intercept | 11.41 (0.30) | <0.001 | 10.80 (0.29) | <0.001 |
| *Class trajectories (Ref: highest memory)* |  |  |  |  |
| 2nd |  |  | -0.32 (0.04) | <0.001 |
| 3rd |  |  | -1.18 (0.05) | <0.001 |
| Lowest memory |  |  | -2.88 (0.09) | <0.001 |
| Age | -0.01 (0.00) | <0.001 | -0.00 (0.00) | <0.001 |
| Female | 0.44 (0.03) | <0.001 | 0.16 (0.03) | <0.001 |
| *Education (Ref: Primary)* |  |  |  |  |
| High school | 0.63 (0.05) | <0.001 | 0.04 (0.05) | 0.35 |
| College or higher | 0.58 (0.04) | <0.001 | -0.01 (0.04) | 0.68 |
| *Marital status (Ref: Single)* |  |  |  |  |
| Married | 0.03 (0.07) | 0.64 | -0.07 (0.07) | 0.33 |
| Divorced | 0.01 (0.09) | 0.86 | -0.10 (0.08) | 0.22 |
| Widowed | -0.77 (0.09) | <0.001 | -0.61 (0.08) | <0.001 |
| *Wealth (Ref: 1^st^ tertile)* |  |  |  |  |
| 2^nd^ tertile | 0.13 (0.04) | 0.002 | 0.00 (0.04) | 0.94 |
| 3^rd^ tertile (richest) | 0.34 (0.04) | <0.001 | 0.05 (0.04) | 0.21 |
|  |  |  |  |  |
| *Smoking behaviour (Ref: Non-smoker)* |  |  |  |  |
| Past smoker | -0.14 (0.03) | <0.001 | -0.11 (0.03) | <0.001 |
| Current smoker | 0.35 (0.06) | 0.001 | 0.34 (0.05) | <0.001 |
| Drinking regularly | 0.17 (0.03) | <0.001 | 0.08 (0.03) | 0.03 |
| Moderate physical activity | -0.57 (0.03) | <0.001 | 0.32 (0.03) | <0.001 |
| Vigorous physical activity | -0.20 (0.04) | <0.001 | 0.09 (0.04) | 0.01 |
|  |  |  |  |  |
| *The presence of chronic diseases* |  |  |  |  |
| Heart diseases | -0.43 (0.08) | <0.001 | -0.30 (0.08) | <0.001 |
| Diabetes mellitus | 0.04 (0.05) | 0.39 | 0.09 (0.05) | 0.07 |
| Stroke | -0.47 (0.08) | <0.001 | -0.30 (0.08) | <0.001 |
| Cancer | -0.22 (0.06) | <0.001 | -0.22 (0.06) | 0.001 |
| Lung diseases | -0.00 (0.07) | 0.97 | -0.05 (0.07) | 0.45 |

**Note:** Coeff=coefficient; SE=standard errors.
