# Supplementary material for: Trajectories of recall memory as predictive of hearing impairment: A longitudinal cohort study
Source: PLoS One. 2020 Jun 18;15(6):e0234623. doi: 10.1371/journal.pone.0234623 (PMC7302912; doi:10.1371/journal.pone.0234623)
Supplement: Attachment — Submitted filename: Response to reviewers2_v(2).docx [file pone.0234623.s006.docx]

Dear Reviewer,

We are very pleased with the review and your efforts in giving feedback on the manuscript. Your recommendations were very useful to strengthen the article. Below you will find your comments numbered and in italic, each followed by a short description of how the comment is implemented in the revised manuscript (in normal font). We have highlighted the changes within the revised manuscripts using coloured text. The number of page and paragraph in our responses refer to the revised manuscripts. We hope that we succeeded in strengthening the paper.

Yours sincerely,

The authors

**Reviewer 1:**

*I am satisfied the authors have responded reasonably to all my prior comments, and I thank them for taking on board my suggestions. I only have the following 3 minor suggestions to make:*

*1) Please check citation [22 – Kiely et al] which examined MMSE change over more than 2 waves of data (4 waves* *spanning 11 years).*

We have revised the citation in the Page 4 lines 79:

‘To our knowledge, only one study has shown that cognitive impairment was associated with faster declines in hearing threshold [22]. That study used data from four waves spanning 11 years of the Mini-Mental State Examination, a verbally administered screen of global cognitive function, to measure the cognitive ability [22].’

*2) Despite being generally sceptical about GMMs I am overall happy with the methods and results as presented – but there are still details about the GMM I would like to see in supplementary table, as this would help make clear how these were estimated. For example, how was the within-class variance modelled? Was variance fixed to be constant within classes, or was it free to vary within classes? A supplementary table showing the full* *parameterisation of the GMM would clarify this.*

In our analysis, we performed latent class growth analysis, in which the variance and covariance estimates for the growth factors within each class are assumed to be fixed to zero. By this assumption, all respondents’ growth trajectories within a class are homogenous. We provide the regression parameters of the latent class models in Supplementary Table 2.

*3) My queries about Figure 1 (why does line end at age 85 end for one class, but extend beyond this range for other classes?) and comments about strong age gradient by trajectory classes were not addressed. Even though these models are adjusted for linear effects of age, I still wonder about the extent to which the substantive finding is driven by age?*

We have added the explanation on Figure 1 in Page 12 lines 220-222:

‘The trajectory of recall memory among the respondents in the highest recall memory group ends at 86 as they are younger than other age groups. The oldest respondent in that group was aged 86 in the final wave.’

**Reviewer 3**

*In ‘Trajectories of recall memory as predictive of hearing impairment: a longitudinal cohort study,’ Maharani and colleagues examine the relationship in later life between cognitive function and subsequent hearing loss. They find that individuals with lower performance on a recall memory task are significantly more likely to develop hearing loss. The manuscript is clearly written and the analysis appear carefully done. A large sample size offsets some weakness in the assessment of hearing loss, which was self-reported at the study onset and based on the HearCheck screener at the final time point. The manuscript should be of interest to readers of PLOS ONE. The main significance of the work is that it provides insight into the directionality of the relationship between cognitive decline and HL (the former potentially leading to the later).*

1. *One aspect of the results that is clear from figure 1 but could be clarified more in the text, throughout the manuscript, is that* *different classes of memory recall trajectory differ more in their starting point or mean than in the rate of decline.*

We have added the explanation on Figure 1 in Page 12 lines 219-220:

‘The recall memory trajectories in Figure 1 differ in the intercepts and mean of the memory scores.’

2. *A second issue that does not appear to be discussed is that the proportion of individuals with objective hearing loss in the study sample appear rather low for this age group, at 6.9%. This could be related to the requirement for HL at 500 Hz, which typically occurs later.*

We have added the discussion on the low prevalence of objective hearing impairment in Page 17-18 lines 320-329:

‘The proportion of the respondents with objective hearing impairment included in this study was lower for the age group (6.9%). Prior studies using national samples showed that the prevalence of hearing impairment among adults aged 50 years and older ranges from 20 to 40% [55]. The definition of hearing loss in this study was not the usual four frequency average of thresholds at 500, 1000, 2000 and 4000 Hz. It was performance on a pure tone audiometry screen with hearing loss equivalent to better ear threshold >35 dB HL at 1000 or 3000 Hz. Hearing loss usually appears first at higher frequencies, so including lower frequencies in an average hearing loss may make the hearing loss index less sensitive to early hearing loss. The 3000 Hz criterion for our hearing loss measure should make it reasonably sensitive to early hearing loss.’

3. *Line 31. It seems important to clarify here that absence of hearing loss was ‘self-reported’*

We have revised the sentence in Page 2 line 31 into:

‘The study population consisted of 3,615 adults aged 50+ who participated in the first wave of the English Longitudinal Study of Ageing, who had no self-reported hearing impairment in Wave 1, and who underwent a hearing test in Wave 7.’

4. *Lines 34-36. Rather than describing model selection here, it might be more useful to sketch out differences in the identified trajectories including different starting points.*

We have added the explanation in the Page 2 lines 37-38:

‘The different recall memory trajectories represent different starting points and mean of the memory scores.’

5. *Line 49. Remove ‘the’ after ‘1989’.*

We have removed the word ‘the’ after ‘1989’ in Page 3 line 50.

6. *Line 113. Do the authors have information on which individuals used hearing aids? It seems like they should be excluded if possible since the objective hearing screening was likely not performed with a hearing aid.*

We are interested in hearing impairment (pathology) indexed by pure tone audiometry test, not functional hearing. There is thus no basis for removing hearing aid users in our study. Excluding hearing aid users would exclude a high proportion of people with audiometric hearing loss, and reduce sensitivity of the analysis.

7. *Line 160. Please clarify what how moderate and vigorous exercise were distinguished. Was there a different question for each?*

We have included the explanation on Page 7 lines 162-164:

‘We categorised respondents as engaging in moderate and vigorous physical exercise if they reported exercising as least once a week. The respondents responded to the questions whether they take part in sports or activities that are vigorous and moderately energetic, respectively.’

8. *Line 194. Please clarify that this was a second analysis.*

We have clarified in Page 9 line 196 that the analysis was the second analysis.

9. *Line 209. It would helpful to have something at the top of the table indicating that ‘Highest’, ‘2nd', etc. refers to the recall trajectory. A header may be missing over ‘Drinking regularly’ since this is not a ‘Smoking Behavior’.*

We have revised the top of Table 1 and included ‘recall memory’ to better explain the recall trajectory. We have also included the header ‘Drinking behaviour’ in the Table.

10. *Line 215. Here is where it would be good to describe the difference in the intercepts/starting points.*

We have added the explanation about the difference in intercepts in the Page 2 lines 37-38:

‘The different recall memory trajectories represent different intercepts and mean of the memory scores.’

11. *Line 217. The decline does not appear ‘linear’ in Fig. 1.*

We have removed the sentence in Page 12 line 219.

12. *Line 238. It would help to indicate the difference between the two columns at the top of the table, i.e., to somehow indicate that the right column includes the recall trajectory. Also, the meaning of the symbols (presumably related to significance) appears to be missing. Finally, the confidence interval for ‘vigorous physical activity’ contains 1 but is marked as significant. Please clarify.*

We have included the information on the different models presented in Table 2, added the information of the symbols and revised the significance for ‘vigorous physical activity’.

13. *Line 255. Noting the different intercepts for the various trajectories would be helpful here. Again, the decline does not appear ‘linear’.*

We have rewritten the sentence in Page 15 lines 257-259:

‘Supporting prior research [33], we identified the highest recall memory trajectory and the lowest recall memory trajectory over time.’
